# Genetic analysis of two Indian families affected with congenital hereditary endothelial dystrophy: two novel mutations in *SLC4A11*

**Published:** 2007-01-16

**Authors:** Arun Kumar, Soma Bhattacharjee, Durgappa Ravi Prakash, Chethan Sitarampur Sadanand

**Affiliations:** 1 Department of Molecular Reproduction, Development and Genetics, Indian Institute of Science, Bangalore, India; 2 Minto Ophthalmic Hospital, Bangalore, India

## Abstract

**Purpose:**

The autosomal recessive form of congenital hereditary endothelial dystrophy (CHED2) is a rare eye disorder caused by mutations in the *SLC4A11* gene located at the CHED2 locus on chromosome 20p13-p12. The purpose of this study was to carry out genetic analysis of CHED2 in two Indian families.

**Methods:**

Blood samples were collected from individuals for genomic DNA isolation. In order to see if these families had mutations in the *SLC4A11* gene, we selected 11 microsatellite markers from the CHED2 candidate region and used them to genotype the families. DNA sequence analysis was used for mutation detection. Allele-specific PCR was used to determine the segregation of mutations in families and also to determine if the mutations were present in 100 ethnically matched normal control chromosomes.

**Results:**

Haplotype analysis suggested linkage of the disorder to the CHED2 locus in both families. DNA sequence analysis showed a novel indel mutation, c.859_862delGAGAinsCCT (E287fsX21) in exon 8 of the *SLC4A11* gene in one family. This mutation is predicted to truncate the protein with a lack of all 14 transmembrane domains. DNA sequence analysis of the second family showed a novel in-frame deletion mutation c.2014_2016delTTC or 2017_2019delTTC which will lead to the loss of a phenylalanine residue at position 672 or 673 (F672del or F673del). The mutant protein is expected to lack a conserved phenylalanine residue in transmembrane domain number 8.

**Conclusions:**

This study reports two novel mutations in two CHED2 families and increases the spectrum of mutations in *SLC4A11* to a total of 16. PCR-based screening methods were developed for both mutations for rapid screening of individuals.

## Introduction

Congenital hereditary endothelial dystrophy (CHED) is a rare corneal genetic disorder. It can be inherited as an autosomal dominant (CHED1, OMIM 121700) or autosomal recessive (CHED2, OMIM 217700) trait in families. It is characterized by diffuse bilateral corneal clouding with no other cause and nystagmus [1]. CHED2 manifests at birth or within the neonatal period and is generally more severe than CHED1, which usually develops later in childhood [1]. CHED1 has been mapped to the pericentromeric region of chromosome 20p11.2-q11.2 [2]. Homozygosity mapping has been implemented to map CHED2 to a different region on chromosome 20p13-p12 in an 8 cM region between D20S113 and D20S882 markers in a large consanguineous Irish family [1]. Mohamed et al. [3] subsequently confirmed the linkage of CHED2 in another family. Recently, Vithana et al. [4] found mutations in the *SLC4A11* gene (also known as *BTR1*, Bicarbonate Transporter-Related protein-1) in 10 CHED2 families from Myanmar, Pakistan, and India. Subsequently, Jiao et al. [5] found mutations in 12/16 Indian families. The SLC4A11 gene has 19 exons, the first and parts of exons 2 and 19 being noncoding [6]. The gene transcribes to an mRNA (GenBank NM_032034) of 3,138 bases, which codes for a protein of 891 amino acids with a calculated molecular mass of 100 kDa [6]. It contains 14 transmembrane domains (TMDs) and intracellular NH_2_- and COOH-termini [6]. It also contains multiple intracellular phosphorylation sites and two extracellular N-glycosylation sites [6]. The gene is expressed in several organs and tissues including eye, blood, lung, ovary, colon, mouth, embryonic tissue, pancreas, kidney, skin, cranial nerve, ascites, prostate, and brain (NCBI Unigene expression profile). Sodium bicarbonate transporter-like solute carrier family 4 member 11 (SLC4A11) is a member of the SLC4 family of bicarbonate transporters [7]. In humans, SLC4 and SLC26 families are the main bicarbonate transporters [7]. SLC4A11 is a Na/borate cotransporter and stimulates cell growth and proliferation by increasing intracellular borate and activating the MAPK pathway [8,9]. Here we report genetic analysis of two CHED2 families ascertained from a south Indian state of Karnataka.

## Methods

### Families

**Figure 1:**
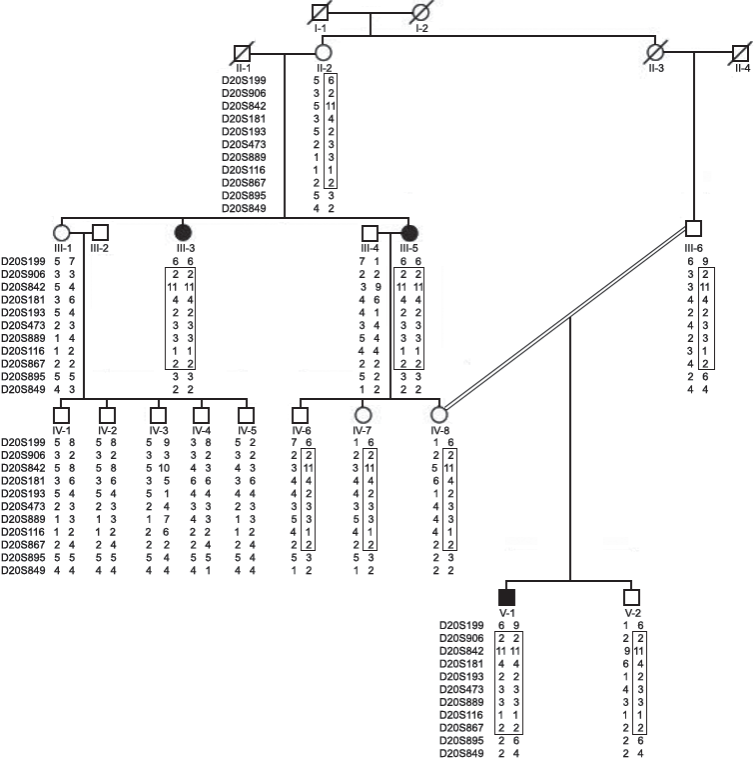
Haplotype analysis of family 1. Haplotype analysis was carried out using 11 microsatellite markers. Disease haplotype is boxed. Note the affected individuals (III-3, III-5, and V-1) are homozygous for the disease haplotype. Individuals II-2, III-6, IV-6, IV-7, IV-8, and V-2 are heterozygous for the disease haplotype and are therefore carriers for the mutation. Empty squares and circles represent normal males and females, respectively. Filled squares and circles represent affected individuals. Affected individuals III-3, III-5, and V-1 are 42, 50, and 6 years old, respectively.

**Figure 2:**
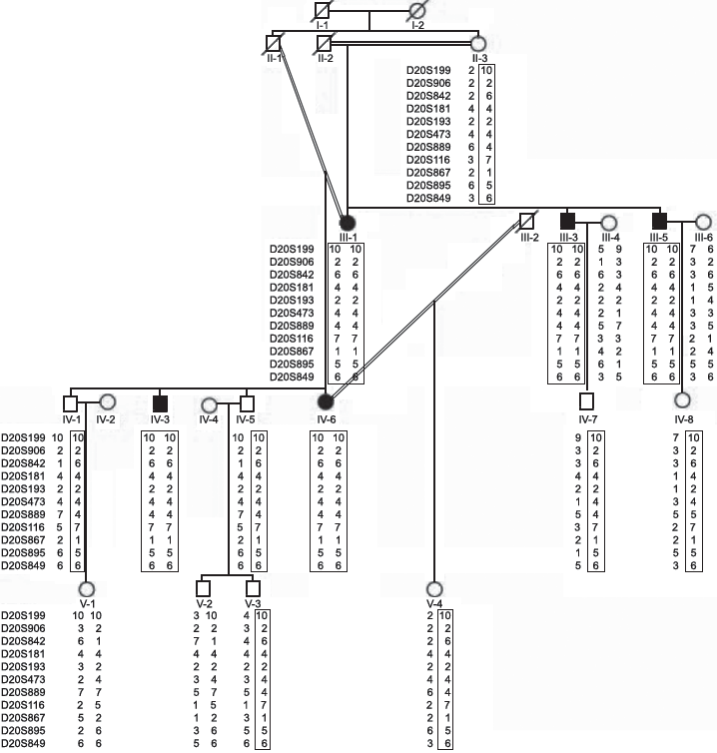
Haplotype analysis of family 2. Haplotype analysis was carried out using 11 microsatellite markers. Disease haplotype is boxed. Note the affected individuals (viz., III-1, III-3, III-5, IV-3, and IV-6) are homozygous for the disease haplotype. Whereas individuals II-3, IV-1, IV-5, IV-7, IV-8, V-3, and V-4 are heterozygous for the disease haplotype and are therefore carriers for the mutation. Empty squares and circles represent normal males and females, respectively. Filled squares and circles represent affected individuals. Affected individuals III-1, III-3, III-5, IV-3, and IV-6 are 70, 65, 45, 30, and 30 years old, respectively.

We have ascertained two multigenerational families (Figure 1 and Figure 2) with autosomal recessive CHED2 from a south Indian state of Karnataka. Family 1 has 17 living individuals including three affected ones. Family 2 has 18 living individuals including five affected ones. All family members were examined in detail. All affected individuals in both families had congenital bilateral cloudy cornea. In family 1, individuals III-3 and III-5 also had secondary spheroidal degeneration of corneal epithelium with nystagmus. Individual V-1 had only nystagmus. All affected individuals from family 2 also had secondary spheroidal degeneration of corneal epithelium with nystagmus. No difference was found in clinical symptoms between families 1 and 2. Informed consent was obtained from each family. Our research followed the guidelines of the Indian Council of Medical Research, New Delhi.

### Genetic analysis

Peripheral blood samples were drawn from individuals in both families into Vacutainer^TM^ EDTA tubes (Beckton-Dickinson, Franklin Lakes, NJ). Peripheral blood samples from 50 ethnically matched and unrelated normal individuals (controls) were also collected. These individuals did not have any signs or symptoms of CHED or any other eye disease. Genomic DNA samples were isolated from peripheral blood samples using a Wizard^TM^ genomic DNA extraction kit (Promega, Madison, WI). To determine if CHED in these families is due to mutations in the *SLC4A11* gene located on chromosome 20p13-p12, we selected 11 microsatellite markers (Table 1) from the CHED2 candidate region [1] and used them to genotype the families. The genotyping was carried out as described in Venkatesh et al. [10]. The haplotypes were constructed by hand. For mutation analysis, we designed primer sets for the entire coding region of the *SLC4A11* gene including intron-exon junctions (Table 2). Mutations in this gene were identified by sequencing the PCR products from one patient from each family on an ABI Prism A310 automated sequencer (PE Biosystems, Foster City, CA) as suggested by the vendor. PCR was carried out in a total volume of 25 μl containing 50-100 ng of genomic DNA, 1.5 mM MgCl_2_, 200 μM of each dNTP, 1X buffer and 1 unit of *Taq* DNA polymerase (Sigma-Aldrich Chemicals Pvt. Ltd., Bangalore, India) using a PTC-100 thermocycler (MJ Research Inc., Waltham, MA). PCR conditions were as follows: An initial denaturation at 95 °C for 2 min was followed by 35 cycles of denaturation at 95 °C for 30 s, annealing at 65 or 67 °C for 30 s, extension at 72 °C for 1 min, with a final extension at 72 °C for 5 min. Prior to sequencing, PCR products were purified using the GenElute^TM^ gel extraction kit (Sigma-Aldrich Chemicals). A mutation was detected in family 1, so all members in this family were examined for the presence of the mutant and wild-type alleles by allele-specific PCR. Allele-specific PCR was carried out using a common reverse primer CHED8R (5'-ACC TCT GGG TGT CTG TGG GCA GGG A-3') and wild-type allele-specific forward primer CHED8WF (5'-GCC TTC CGC CAG AAG CTC CTG GAG A-3') or mutant allele-specific forward primer CHED8MF (5'-GCC TTC CGC CAG AAG CTC CTG CCT C-3'). CHED8WF-CHED8R and CHED8MF-CHED8R primer sets were used to amplify 161 and 160 bp amplicons, respectively with an annealing temperature at 62 °C. Allele-specific PCR was also used to determine if the mutation in family 1 was not present in 50 ethnically matched normal controls. Similarly, after a mutation was detected in family 2, the rest of the family members and 50 controls were examined for the presence of the mutant and wild-type alleles by allele-specific PCR. Allele-specific PCR was carried out using a common reverse primer CHED15R (5'-AGG AGG TCC CAG TGG TAG GCA GTG-3') and wild-type allele-specific forward primer CHED15WF (5'-CTT CCT GCT GTC CAT GCT CTT CTT CA-3') or mutant allele-specific forward primer CHED15MF (5'-GGC TTC CTG CTG TCC ATG CTC TTC A-3'). CHED15WF-CHED15R and CHED15MF-CHED15R primer sets amplified 192 and 189 bp amplicons, respectively with an annealing temperature at 62 °C. Mutation nomenclature is according to den Dunnen and Antonarakis [11].

**Table 1:** Microsatellite markers from the candidate region of the *SLC4A11* gene.

**Number**	**Markers**	**Genetic distance (cM)**
1	D20S199	6.25
2	D20S906	7.61
3	D20S842	8.97
4	D20S181	9.53
5	D20S193	9.53
6	D20S473	9.53
7	D20S889	11.2
8	D20S116	11.2
9	D20S867	12.12
10	D20S895	13.98
11	D20S849	13.98

Genetic distance was obtained from the Marshfield Medical Research Foundation web site. Marker order was established using the sequence map from the UCSC Genome Bioinformatics site. According to this site, the order of markers is as follows: D20S199-D20S906-D20S842-D20S181-D20S193-D20S473-D20S867-D20S889-D20S116-D20S895-D20S849.

**Table 2:** Primers used for the mutation analysis of the *SLC4A11* gene.

**Exon**	**Sequence (5'-3')**	**Annealing temperature (°C)**	**Amplicon size (bp)**
2	F: ATTAAGGCTGGCTTCCCCTGCTATG	67	303
	R: CCCTGGAGGCTTTTGCCCGACAAG		
3&4	F: CCTTCCTGTGTGTGGCACTTTAACAG	67	439
	R: ATCACCTCAGCCCCCAGGTAGAGG		
5	F: ACCAGGCAGTGACAGCATCTCATAC	67	354
	R: GGGTGGTGGGTCAACAGCCCCTC		
6	F: GGGGGCGTTGGGAGGGGCTGTTGA	67	201
	R: AGGGGACATGGGACACCCAGTTCCAC		
7	F: GTCGGGGAGCCCCAGCTCCCTGG	67	254
	R: GGACCCCAAGCAGAGGGCGGGTAA		
8	F: CCCGGGCAGGGCCTCCTCTGTTTC	67	328
	R: GACAGAGCGCCTGTTAGCCCTGTCC		
9&10	F: TCGGCGGGGGCATGGGCCGGACA	67	419
	R: AGCCCAGGGCCCAGCCCAGCATAC		
11	F: TATGCTGGGCTGGGCCCTGGGCTG	67	444
	R: GGGCTGAACCAGATCCCAAGCCTTGA		
12	F: GGGGCTCAGGGAGGCCTCCCCCA	67	210
	R: AGTGCAGAACCTCCCATCTCGGCTG		
13	F: TCCAGGGCCTCCCCCTGCCACAC	67	382
	R: GGGACAGCAGGTGCATGAGCACAGC		
14	F: GAGGGGAGGGGCCGCATGGGTCAA	67	239
	R: AGTAGGGGACAGGCTACTGCTATGCC		
15	F: GGCGGTGGGTGACGTGGGGTAGC	67	304
	R: CTCGTGGACAGAGCCCCACAGCAGA		
16	F: CACCGGAGAACAGGTGTGGAGGGTG	65	325
	R: GGCCAGAGGCTCCCCACTCCTCAG		
17	F: GGAGGAGTGAGGCCCTGTGGACAGG	67	340
	R: TGTGGGCGGCAGGGACCGGGTGTG		
18	F: GGCGTGGGTGGGGACACAGCCCCA	67	289
	R: CAGCCCGCCCATTCTCCACACCTAGA		
19	F: TGGGATGGGTGTCCACTGCCTTCTC	67	199
	R: GCTCCAGAGCCAGCCTGGGAGGAC		

Primers were designed using genomic DNA and mRNA (GenBank NM_032034) sequences from the UCSC Genome Bioinformatics site. This mRNA does not have exon 1 sequences. Exon 1 was not analyzed as it is a part of the 5'-UTR.

## Results & Discussion

Visual inspection of both pedigrees (Figure 1 and Figure 2) suggested that CHED is segregating as an autosomal recessive trait in both families. Haplotype analysis using 11 microsatellite markers selected from the candidate region of the CHED2 locus showed that all the affected individuals in family 1 were homozygous for D20S906, D20S842, D20S181, D20S193, D20S473, D20S867, D20S889, and D20S116. Heterozygosities at D20S199 and D20S895 in affected individual V-1 placed the CHED2 locus between D20S199 (6.25 cM) and D20S895 (13.98 cM) markers in a genetic distance of 7.73 cM. A disease haplotype 2-11-4-2-3-3-1-2 at marker loci D20S906-D20842-D20S181-D20S193-D20S473-D20S867-D20S889-D20S116 cosegregated with the disease in family 1 (Figure 1). Haplotype analysis using the same set of markers showed that all affected individuals in family 2 were homozygous for all 11 markers (Figure 2). A different disease haplotype 10-2-6-4-2-4-4-7-1-5-6 co-segregated with the disease at marker loci D20S199-D20S906-D20842-D20S181-D20S193-D20S473-D20S867-D20S889-D20S116-D20S895-D20S849 in family 2 (Figure 2). This suggested that the CHED phenotype in both families is linked to the CHED2 locus, and different mutations are responsible for the disease phenotype in both families. Recently, Vithana et al. [4] identified the gene responsible for CHED2; it lies between D20S181 and D20S193. Therefore, we directed our efforts in the mutation analysis of the *SLC4A11* gene in both families.

**Figure 3:**
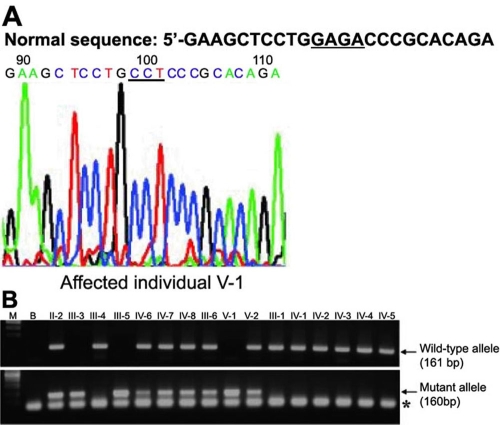
Mutation analysis of the *SLC4A11* gene in family 1. **A**: Sequencing chromatogram of the exon 8 PCR product from the affected individual V-1. Normal DNA sequence is written on the top of the chromatogram. The deleted nucleotide residues "GAGA" is underlined in the normal sequence. Note deletion of GAGA and insertion of CCT (underlined) in the chromatogram. **B**: Agarose gel electrophoresis of PCR products from all individuals using wild-type (upper panel) and mutant allele-specific primer sets (lower panel). Note all normal individuals had bands in the upper panel as expected. All heterozygotes and patients had bands in the lower panel as expected. Lanes M and B stand for 100 bp marker and no template PCR, respectively. Individuals noted on top of the gel pictures are as in Figure 1. An asterisk marks the primer-dimer band.

DNA sequence analysis of the affected individual V-1 from family 1 showed a deletion of four nucleotide residues GAGA from nucleotide positions 859 to 862 in exon 8, which was replaced by an insertion of three nucleotide residues CCT in a homozygous state (Figure 3A), resulting in an indel mutation c.859_862delGAGAinsCCT. As expected from the haplotype data (Figure 1), allele-specific PCR showed that all patients and heterozygotes had the mutant allele (Figure 3B). The wild-type allele was present in heterozygotes and normal individuals (Figure 3B). This mutation was not present in 100 normal control chromosomes (data not shown). This mutation is predicted to truncate SLC4A11 protein with the introduction of 21 novel amino acid residues beginning from amino acid position 287 (E287fsX21). The mutant protein is predicted to lack all 14 transmembrane domains. This suggests that the disease phenotype could be due to the lack of the mutant protein in the membrane.

**Figure 4:**
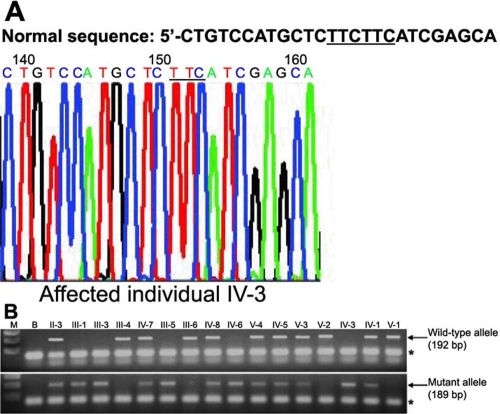
Mutation analysis of the *SLC4A11* gene in family 2. **A**: Sequencing chromatogram of the exon 15 PCR product from the affected individual IV-3. Normal DNA sequence is written on the top of the chromatogram. The tandem repeats of two "TTC" are underlined. Note only one TTC (underlined) in the chromatogram. **B**: Agarose gel electrophoresis of PCR products from all individuals using wild-type (upper panel) and mutant allele-specific primer sets (lower panel). All normal individuals have bands in the upper panel as expected. All heterozygotes and patients have bands in the lower panel as expected. Lanes M and B stand for 100 bp marker and no template PCR, respectively. Individuals noted on top of the gel pictures are as in Figure 2. An asterisk marks the primer-dimer band.

DNA sequence analysis of the affected individual IV-3 from family 2 revealed in-frame deletion of three nucleotide residues TTC from nucleotide positions 2014 to 2016 or 2017 to 2019 as there are two tandem TTC residues in exon 15 in a homozygous state, resulting in the mutation c.2014_2016delTTC or c.2017_2019delTTC (Figure 4A). As expected from the haplotype data (Figure 2), allele-specific PCR showed that all patients and heterozygotes had the mutant allele (Figure 4B). The wild-type allele was present in heterozygotes and normal individuals (Figure 4B). This mutation was not present in 100 normal control chromosomes (data not shown). This mutation will lead to the loss of a phenylalanine residue at position 672 or 673 (F672del or F673del). Phenylalanine residues at position 672 and 673 lie in the transmembrane domain number 8 of the SLC4A11 protein [6]. This suggests that the deletion of phenylalanine at either position may disrupt the localization or proper assembly of this protein in the membrane. It is possible that the true functional polymorphism (mutation) may not be this deletion and rather another polymorphism in LD (linkage disequilibrium) with the deletion. However, this possibility is unlikely as no other mutation has been detected in the entire coding region and at intron-exon junctions of this gene in the affected individual IV-3. Moreover, the phenylalanine residue at position 673 is conserved in members of the human bicarbonate transporter superfamily such as AE4, NBC1, NDCBE1, AE1, and CeBTR [6]. Interestingly, in-frame deletion of a phenylalanine residue at position 508 (deltaF508) has been reported in another transmembrane protein CFTR [12]. However, the deletion of phenylalanine residue is not located in TMD and occurs in the nucleotide ATP-binding fold 1 (NBF1) region of CFTR [12].

Vithana et al. [4] reported a total of seven mutations in this gene, which include four missense, one nonsense, one deletion, and one acceptor site splice mutations in 10 CHED2 families from Myanmar, Pakistan, and India (Table 3). Jiao et al. [5] carried out mutation analysis of the *SLC4A11* gene in 16 Indian families and found nine different mutations in 12 families; four missense, two nonsense, one deletion and two indel mutations. Two mutations, R605X and R755Q, were identified by both groups (Table 3). When data from both groups and the present study are considered together, the total number of mutations in the *SLC4A11* gene reaches 16 which includes seven missense, two nonsense, three deletions, and four indel mutations (Table 3).

**Table 3:** Summary of mutations detected so far in the *SLC4A11* gene in CHED2 families.

**Mutation**	**Exon/intron**	**Nature of mutation**	**Effect of protein**	**Ethnic origin of families**	**Reference**
g.2943delTTinsA (R82RfsX33)	2	Indel	Truncation of protein and addition of novel amino acids	1 Indian family	[5]
c.353_356delAGAA	4	Deletion	Truncation of protein	1 Indian family	[4]
c.859-862delGAGAinsCCT (E287fsX21)	8	Indel	"Truncation of protein and addition of novel amino acids, absence of all TMDs"	1 Indian family	Present study
c.1391G>A (G464D)	11	Missense	Conformation change	3 Pakistani families	[4]
c.1466C>T (S489L)	12	Missense	Conformation change	1 Pakistani family	[4]
g.8118delCT (H568HfsX177)	13	Deletion	Truncation of protein and addition of novel amino acids	1 Indian family	[5]
c.1813C>T (R605X)	14	Nonsense	Truncation of protein	3 Indian families	[4], [5]
g.8379G>T (E632X)	14	Nonsense	Truncation of protein	1 Indian family	[5]
IVS15-6_-16delins GGCCGGCCGG	15	Indel	Inactivation of an accepter splice site	1 Indian family	[4]
c.2014_2016delTTC or c.2017_2019delTTC (F672del or F673del)	15	In-frame deletion	Disruption of TMD number 8	1 Indian family	Present study
c.2264G>A (R755Q)	17	Missense	Conformation change	2 Indian and 1 Myanmar families	[4], [5]
g.9191G>A (R804H)	17	Missense	Conformation change	1 Indian family	[5]
g.9200delTinsGG (L807RfsX71)	17	Indel	Truncation of protein and addition of novel amino acids	1 Indian family	[5]
g.9361C>T (T833M)	18	Missense	Conformation change	2 Indian families	[5]
c.2605C>T (R869C)	18	Missense	Conformation change	1 Indian family	[4]
g.9469G>A (R869H)	18	Missense	Conformation change	2 Indian families	[5]

A total of 16 *SLC4A11* mutations have been detected so far in 24 CHED2 families from Myanmar, Pakistan, and India.

In summary, we report two novel mutations (c.859_862delGAGAinsCCT/E287fsX21 and c.2014_2016delTTC or c.2017_2019delTTC/F672del or F673del) in the *SLC4A11* gene in two Indian CHED2 families. Of 24 CHED2 families with mutations in *SLC4A11*, 23 are from India (Table 3). It will be interesting to see the range and types of mutations in this gene in families from other populations. We suggest that the disease phenotype in both families could be due either to the absence of the protein in the membrane or improper membrane localization of the protein. The present information will be useful to provide rapid prenatal diagnosis and genetic counseling to families and their relatives using an allele-specific PCR method developed during our study.
